# Progress in Neurology

**Published:** 1902-06-21

**Authors:** 


### Progress in Neurology.

**Tabes.**—In a most useful paper Bramwell[1] praises Frenkel's treatment of the ataxy of tabes and gives precise instructions as to how it is carried out. Frenkel pointed out in 1889 that in cases of tabes by the accurate repetition of a movement a notable diminution of the ataxia in association with that movement is produced, and this observation has been abundantly confirmed. The method is indeed one of re-education. The amount and rapidity of the improvement is largely influenced by the personal equation of the patient; the best results are obtained in persons who take an intelligent interest in the exercises and who are gifted with mental concentration and perseverance. The results are often striking; thus, a man who had been unable to walk or stand for three years was, after ten weeks' treatment, able to walk alone with a stick. A notable change in the patient's mental disposition often occurs; from being depressed and miserable he becomes again hopeful and cheerful, owing to the improvement in the ataxy. The patient should be told that it is not the muscular force which he uses in performing the exercises which is of importance, but the care and precision with which he makes the individual movements. The exercises for the lower limbs are as follows: The patient lies on his back in bed and slowly raises his extended leg until he touches with his great toe the finger of the attendant held immediately above his foot, at a distance of 18 inches to 2 feet from the bed. Still lying on his back, he flexes the leg on the thigh to its full extent, and then the thigh on the abdomen, the whole limb is gradually extended until he touches with his great toe the finger of the attendant, which is held in the same position as in the previous exercise. Standing exercises are carried out by the patient being supported on either side, and then encouraged to practise his balancing power, gradually putting more and more weight upon his legs; he is also to practise standing with his feet close together, lifting one foot off the ground and placing it down again accurately. Walking exercises are performed by painting a black stripe 12 inches broad across the floor of a room, and along this the patient walks with support, taking care to keep his feet within its margins. Having mastered this exercise, he practises walking along a similar stripe on which, at distances of 1 foot, cross lines are painted, taking care to put his toe down exactly at the cross line. When these exercises are performed accurately, the strip may be made narrower—6 inches in breadth. Again, the patient must practise sitting down and getting up from his chair, walking up and down stairs, and so on. A quarter of an hour, two or three times a day, may be given to these exercises, and after each exercise the patient should take a short rest. It should be stopped on the earliest appearance of fatigue, or if there are any signs of the patient's attention beginning to wander.

**Treatment of Hemiplegia.**—Guthrie[2] says that cases of hemiplegia are often not treated from the opinion that absolutely nothing can be done for them. Early and long-continued treatment, however, can do much—both hasten the recovery of mild cases and retard the absolute helplessness of severe ones. Thus, articular adhesions frequently cause limitation of movement. They may occur in the elbow, wrist, hip, knee, and ankle joints, but are most common, and are formed earliest, in the shoulder, rendering movements of the joint painful, if not impossible; and a limb which might have recovered is often useless owing to their presence. These adhesions can be prevented by passive movements of each joint from the first. Early rigidity, which so often limits movement, can be partially counteracted by stimulation of the opponents of the spastic muscles by massage. Thus, in the arm, the adductors of the shoulder, flexors of the elbow, pronators of the forearm, flexors of the thumb and fingers, adductor and opponens muscles of the thumb, are usually contracted, and should be left alone, whilst their opponents are regularly massaged and kept in good condition. Electricity, whether faradaism or galvanism, is a useful adjunct to massage, but cannot take its place. Re-education of movements is very necessary. A patient may not move his limbs, not so much because they are paralysed, but because he has forgotten how to do so. He must be taught, by executing the movement passively so that he may see how it is done, and getting him to try himself. Recovery naturally takes place first in the least specialised movements. The patient must therefore be taught to sit up before he can stand, stand before he can walk, move his shoulder before his elbow, and so on. Want of precision in movement often follows recovery, the regained movements are ataxic. Exercises on the Frenkel system, as employed in locomotor ataxy, are useful in such cases. This treatment of hemiplegia can be adopted quite irrespective of its cause, and need not interfere with other measures taken for the relief of the disease which gave rise to the hemiplegia.

[1] Edin. Med. Jour., Sept. 1901. [2] Lancet, Oct. 19, 1901.

